# A national-level analysis of life expectancy associated with the COVID-19 pandemic in India

**DOI:** 10.3389/fpubh.2022.1000933

**Published:** 2022-10-18

**Authors:** Malaisamy Muniyandi, Pravin Kumar Singh, Yamini Aanandh, Nagarajan Karikalan, Chandrasekaran Padmapriyadarsini

**Affiliations:** ^1^Department of Health Economics, ICMR-National Institute for Research in Tuberculosis, Chennai, India; ^2^Department of Social-Behavioral Research, ICMR-National Institute for Research in Tuberculosis, Chennai, India; ^3^Department of Clinical Research, ICMR-National Institute for Research in Tuberculosis, Chennai, India

**Keywords:** COVID-19, life expectancy, longevity, mortality, India

## Abstract

**Background:**

From a demographic perspective, the impact of severe acute respiratory syndrome coronavirus 2 (SARS-CoV-2) on life expectancy is not clear. Hence, there is a need to study the number of years of life lost concerning the existing average life expectancy due to COVID-19 in India.

**Objective:**

This study aimed to estimate the impact of life expectancy due to the COVID-19 pandemic in India.

**Methodology:**

We considered day-wise age-specific mortality due to COVID-19 which was extracted from the COVID-19 data repository from March 11, 2020, to June 30, 2021, in India. All-cause mortality was collected from the United Nations population estimates. An abridged life table technique was utilized for calculating life expectancies based on all-cause mortality and mortality due to COVID-19. MortPak software was used to calculate the life expectancy with and without the COVID-19 pandemic. Life expectancy at birth in different age groups was estimated with respect to with and without COVID-19.

**Results:**

A total of 399,459 deaths due to COVID-19 were distributed age wise, and their corresponding life expectancy was calculated. The general mortality was compared with COVID-19 mortality for the various age groups, and it was observed that mortality due to COVID-19 was significantly higher among the elderly age group [i.e., 45 to 60 years (36%) and > 60 years (51%)] when compared with < 25 years (1%) and 26–44 years (11%) (trend Chi-square 7.59; *p* = 0.001). The life expectancy without and with COVID-19 was 69.28 years and 69.16 years, respectively.

**Conclusion:**

Overall, it was estimated that COVID-19 has an impact on life expectancy by 0.12 years during the study period. Even though mortality due to COVID-19 was high, factors such as lockdown, vaccination, and accidents also had an influence on mortality. Thus, there is a need to assess the impact of COVID-19 on life expectancy in future.

## Introduction

Severe acute respiratory syndrome coronavirus 2 (SARS-CoV-2) emerged in Wuhan, Hubei, China, in late December 2019 (1) and was declared a pandemic by the World Health Organization (2). The SARS-CoV-2 belongs to the family of SARS and is the cause of respiratory disease known as coronavirus disease 2019 (COVID-19). The disease spreads from person to person through small droplets of an infected person's cough or sneeze, and these can settle down on the nearby object or area and can make it infectious. Symptoms including fever, cough, and pneumonia range from mild to severe (2). In addition to affecting public health, the COVID-19 pandemic globally sparked a severe demographic and socioeconomic crisis (2). The COVID-19 pandemic affected several countries, and it was the greatest threat to life expectancy, resulting in an unprecedented rise in mortality caused by COVID-19 and significant years of life lost.

Documented fatality due to COVID-19 from South Asia was 1% (3, 4). In India, the first COVID-19 case was documented on January 27, 2020, in Kerala (5). India is the second most populous country in the world with a population of 1.35 billion, but India had a slow pace of COVID-19 spread over the first three months, from January 2020 to March 2020. A total of 399,459 deaths due to COVID-19 were reported from March 11, 2020, to June 30, 2021. Though India had the lowest mortality rate, it also has the highest recovery rate for COVID-19.

Life expectancy at birth is a commonly used indicator and a key summary measure of the health and well-being of the population. It is the widely used metric of population health and longevity. It refers to the average number of years a hypothetical cohort of people would live if they were to experience the death rates observed in a given period throughout their lifespan. The life expectancy is estimated based on the death rates for a given period. Over a period, life expectancy at birth increased significantly in most countries. It was reported that people in developed countries have higher life expectancy than others (6). Improvements in life expectancy among high-income countries were predominantly driven by gains made at older ages (7). Few countries experienced significant gains in life expectancy in the past decade (8), whereas other countries witnessed noticeable slowdowns in the pace of improvements and some countries stalls or temporary reversal (9). People with the COVID-19 infection are more prone to many life-threatening morbidities and mortalities. Almost 1.8 million estimated lives have been lost due to COVID-19 around the globe in 2020 (10, 11). The COVID-19 pandemic has resulted in a change in the life expectancy of the population. COVID-19 mortality experience in the USA, Italy, North America, Europe, Colombia, Canada, and Liberia suggests that it has a major impact on 2020 life expectancy (12). The life expectancy losses are viewed as a cause of concern, and actual declines in life expectancy are alarming.

During the COVID-19 pandemic, life expectancy progress became more varied, and the pandemic triggered a global crisis posing additional challenges to population health. Mortality due to COVID-19 was higher in all age groups, and it was higher in females than in males and higher among the older population (13). In addition, the pandemic also indirectly increased other causes of death due to delay in diagnosis and treatment. Population-level studies on the impact of the COVID-19 pandemic through life expectancy loss were carried out in 29 countries (12). It was reported that the USA and Eastern European countries such as Lithuania, Bulgaria, and Poland experienced significant losses in life expectancy in 2020 (12).

From India, Suryakant Yadav et al. (14) reported the inequality of life expectancy between males and females and in different age groups using the Gini coefficient; Guru Vasishtha et al. (15) reported the impact of COVID-19 infection on life expectancy, premature mortality, and DALY from one state of India; and Jha et al. (16) reported the comparison of officially reported and estimated COVID-19 deaths. We do not have a population-level study on life expectancy for the entire country. Hence, this current study examines the impact of the COVID-19 pandemic on life expectancy in 2020 specific to India. We focused on the change in life expectancy in 2020 relevant to COVID-19.

## Methods

### Study setting

India is the second most populated country in the world accounting for one-sixth of the world's population. The present population of India in 2022 is 1,417,173,173, and the median age was 28.7 years. It is expected to become the first country to be home to more than 1.5 billion people by 2030. For people living in a resource-poor country with a high population density, planners recognized that population stabilization is an essential prerequisite for sustainable development. As per the National Population Policy, India has set the goal of achieving population stabilization by 2045. During the last five decades, there have been a sharp decline in mortality and a sustained decline in fertility. Recent trends show that India's population growth has already peaked and it is on the decline now. The total fertility rate declined significantly from 3.4 in 1993 to 2.2 in 2016 as per the National Family Health Survey 2015–2016. The ongoing global pandemic of COVID-19, which started at the end of 2019, and outbreaks have caused a significant number of deaths worldwide including India. Since then, India is one of the countries experiencing excess mortality caused by COVID-19 and has more than 10.3 million confirmed cases, and the case fatality rate was 1.4% (17). India ranks the third position globally in terms of deaths attributable to COVID-19 in 2020.

### Source of data

Data for this study were collected from different secondary sources. These include the United Nations population estimates, the United Nations World Population Prospects, and the COVID-19 repository of the Center for Systems Science and Engineering (CSSE) at John Hopkins University. An age-specific all-cause mortality rate for the country for the year 2020–2021 was taken from the United Nations population estimates prepared by the Population Division of the Department of Economic and Social Affairs of the United Nations Secretariat (18). Information on the COVID-19-confirmed cases and deaths by age group and day wise was collected from the COVID-19 repository of the CSSE at John Hopkins University. The distribution of deaths due to COVID-19 provided by the COVID-19 repository of the CSSE was verified with COVID-19-India Application Programming Interface (API), Ministry of Health and Family Welfare, Government of India, portal available in the public domain (19). The estimated average years of life expectancy for India were collected from the *macrotrends* provided by the United Nations World Population Prospects from 1955 to 2020 (20).

### Construction of life tables

A life table is a mathematical model that portrays mortality conditions at a particular time among a population and provides a basis for measuring longevity. It is based on age-specific mortality rates observed for a population for a particular year (21). In this current study, an abridged life table technique was used to calculate life expectancies based on all-cause mortality and mortality due to COVID-19. We formulated the following seven different columns to construct the life table: (1) an age-specific interval or period of life between two exact ages stated in years *(x, x*+ *n)*. (2) the proportion of persons alive at the beginning of the age interval who die during the age interval (_*n*_*q*_*x*_). (3) the starting number of new born in the life table called the radix of the life table, usually set at 100,000, the number living at the beginning of the age interval, or the number surviving to the beginning of the age interval *(l*_*x*_). (4) the number of persons in the cohort who die in the age interval *x, x*+ *n (*_*n*_*d*_*x*_). (5) the number of years of life lived by the cohort within the indicated age interval *x, x*+ *n* or person-years of life in the age interval (_*n*_*L*_*x*_). (6) the total person-years of life contributed by the cohort after attaining age x *(T*_*x*_), and (7) the average number of years of life remaining for a person alive at the beginning of age interval x *(e*${}_{x}^{0}$).

### Data analysis and statistical tools used

Data were entered in MS Excel. MortPak software (version 4.3) was used to estimate life expectancy at birth as well as for every 5-year age group at birth 0–1, 1–5 to 95—years. Trend Chi-square test was used to compare the life expectancy with and without COVID-19 at a 5% level of significance.

This study is merely based on a type of mathematical computation called the “differential method.” The differences in life expectancy can be explained based on observed changes in mortality (22).

### Time period

We measured the variation of life expectancy in different age groups for the general population and the COVID-19-affected population from March 11, 2020, to June 30, 2021, for the first and the second waves.

### Ethical considerations

This study considered secondary data which were collected from data repositories which are freely available in the public domain. Hence, ethical clearance is not required.

## Results

It was observed that overall life expectancy at birth was 69.28 years (Supplementary Table S1). Life expectancy at birth will decrease as age increases (70.56 to 4.05 years). Life expectancy in different age groups was calculated (Supplementary Table S2) based on 399,459 deaths due to COVID-19.

### Comparison of general mortality with mortality due to COVID-19

**Figure 1:**
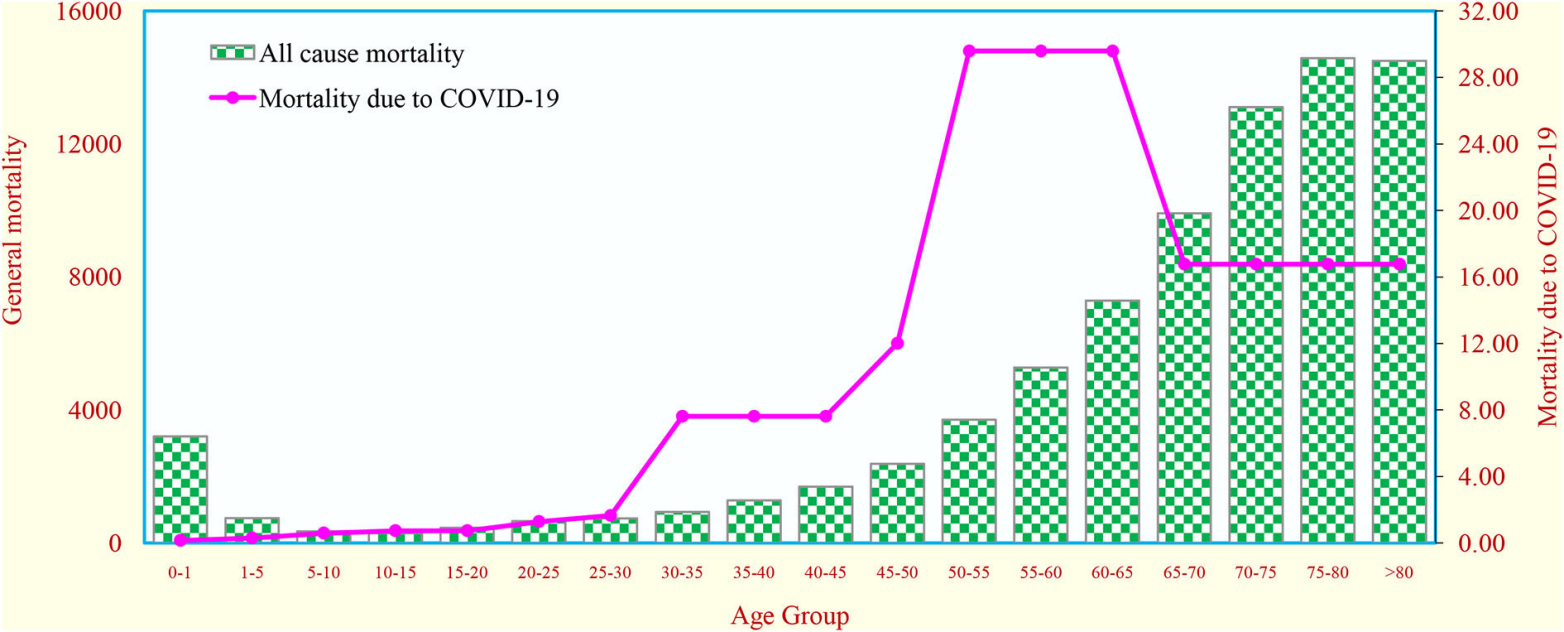
Comparison of probability of death due to all–cause mortality with probability of death due to COVID−19 in different age groups in India.

The general mortality was compared with COVID-19 mortality for the various age groups (Figure 1). It was observed that the mortality due to COVID-19 was significantly higher among the elderly age group [i.e., 45 to 60 years (36%) and > 60 years (51%)] when compared with < 25 years (1%) and 26–44 years (11%) (trend Chi-square 7.59; *p* = 0.001). The results highlighted that COVID-19 deaths significantly increase as age increases (Supplementary Figure S1).

### Comparison of life expectancy at birth

We generated the abridged life table to compare life expectancy at birth for both all-cause mortality and COVID-19 mortality (Supplementary Table S3). It was observed that there was a high mortality due to COVID-19 (0.39%). There was a decline of 43 days in life expectancy due to COVID-19 mortality (Supplementary Table S4). The life expectancy without and with COVID-19 was 69.28 years and 69.16 years, respectively.

### Comparison of age-specific life expectancy

**Figure 2:**
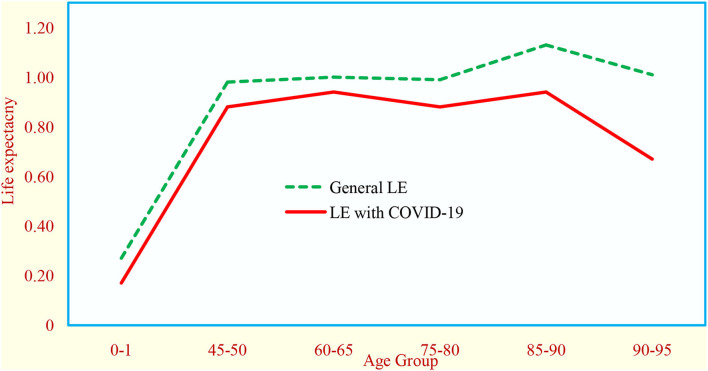
Effect of COVID−19 on life expectancy at birth in India (June 2020 estimates).

We compared the life expectancy with and without COVID-19 in various age groups (Supplementary Table S4). The life expectancy with COVID-19 in 0–1 year, 45–50 years, 60–65 years, 75–80 years, 85–90 years, and more than 90 years was 0.28, 0.98, 1.03, 0.99, 1.14, and 1.05 years, respectively, whereas the life expectancy without COVID−19 in 0–1 year, 45–50 years, 60–65 years, 75–80 years, 85–90 years, and more than 90 years was 0.16, 0.87, 0.93, 0.87, 0.92, and 0.64 years, respectively. The difference between the life expectancy with and without COVID−19 in 0–1 year, 45–50 years, 60–65 years, 75–80 years, 85–90 years, and more than 90 years was 0.12, 0.11, 0.10, 0.12, 0.22, and 0.41 years, respectively (Figure 2).

### Life expectancy in India over a period

**Figure 3:**
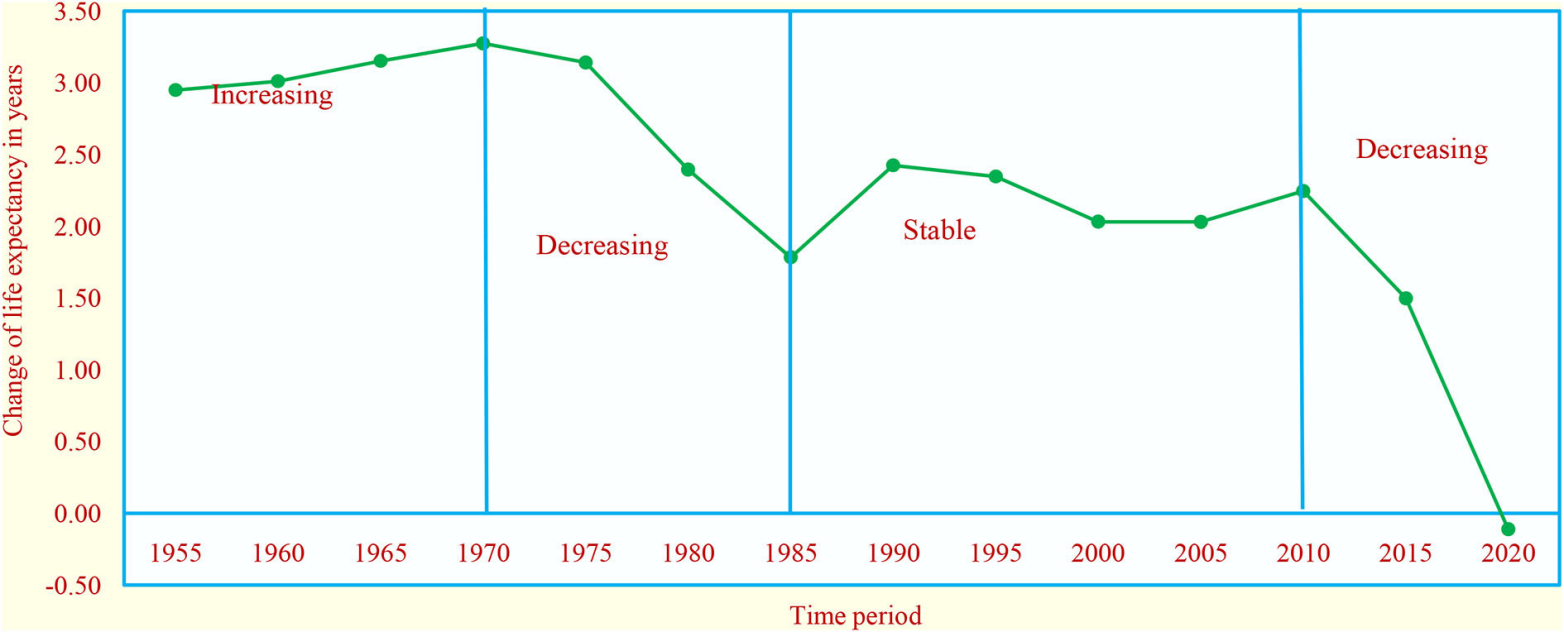
Years of life expectancy change in five–year intervals in India.

From 1950 to 2021, life expectancy at birth increased from 36.98 to 69.28 years, while a gain of 32.3 years seems to be a significant increase, which represents a decrease in rate over a period (Supplementary Figure S2) in India. From 1950 to 1970, there was an increasing trend (average 3.1 years), there was a decreasing trend during 1970–1985 (average 2.6 years), it was almost stable during 1985–2010 (average 2.1 years), and then, there was a decreasing trend (average 1.9 years) (Figure 3) in the subsequent years.

## Discussion

The current study has documented the national–level analysis of life expectancy associated with the COVID−19 pandemic based on 399,459 reported deaths from 2020 to 2021. This study found that there is a decrease in life expectancy by 43 days (0.12 years) due to COVID−19. Similar studies from developed countries reported that those who were severely affected by COVID−19 have also lost significant years of life. It was estimated that there is a decline in life expectancy in Canada by 0.41 years (23), in Italy by 0.5 years (12), and in Columbia by 0.18 (12) years.

The other salient finding from our study was that there was a decrease in life expectancy in all the age groups. However, increased mortality among the elderly age group (i.e., more than 60 years) resulted in a decrease in life expectancy during the pandemic. A similar finding reported that more than one–third of COVID−19 deaths were in the age group of more than 65 years (24). As COVID−19 caused an excessive number of deaths in the elderly population, the number of years lost concerning the existing average life expectancy might be smaller than expected. As long as the prevalence does not exceed a particular threshold, the loss in life expectancy is minimal. With a prevalence rate of < 1%, the years of life lost are anticipated to be less than the yearly secular increase, which is approximately 0.2 in high–income countries. It is a known fact that the increase in mortality will result in a decline in period life expectancy. It was documented that in North America and Europe, the loss in period life expectancy would vary from 4 to 11 years at very high mortality due to COVID−19 (25, 26). In India, Maharashtra was significantly affected by the COVID−19 pandemic and the impact on life expectancy at birth was estimated, and it is likely to reduce by 1.4 years (12). This was higher as compared to our current national–level estimate.

It is a well–known fact that as the age increases, there will be a considerable reduction in life expectancy. More than 60% of COVID−19 cases are in the age group of 30–64 years which is the economically productive segment of the population. From an economic perspective, health and longevity are very consequential. Health is believed to drive economic growth due to a healthier workforce that is more productive. The pandemic will have short– and long–term implications on socioeconomic determinants of health. In addition, a demographic perspective of the impact of COVID−19 on life expectancy also should be considered, as the mortality in a particular age group of the population may affect the structure of the population.

During the first wave due to the national lockdown and other preventive measures, there was not much death reported. Currently, India is facing the fourth wave, and the last two waves severely affected the country concerning the number of COVID−19 infections, where the disease remains a constant threat due to a large number of population, cities with compact settings, and inadequate healthcare facilities. The consequences of the COVID−19 pandemic, particularly in terms of mortality, will be reduced eventually by the existing interventions such as vaccination, XraySetu (AI–driven platform) for early identification of COVID−19 positivity, and rapid antigen test (RATs) COVID−19 home testing kit.

The life table technique is used by actuaries, demographers, and many others to study reproduction, migration, fertility, and population growth. It is also used to make a relative comparison of various measures of mortality such as death rate and expectations of life for two or more different groups of the population. The life table is accepted widely as an important tool in demographic and public health studies. In this current study, we used the life table technique to measure the impact of COVID−19 on life expectancy in India.

### Limitation

In this study, we are not able to calculate gender–specific life expectancy due to the non–availability of data. We employed data for a single cross section of the period from March 2020 to June 2021 to describe the current mortality pattern due to COVID−19. They are officially reported death data, but not actual death data. The main advantage is that it provides measures localized in time. Also, the data pertain to a limited period.

The life expectancy in India overall seemed to be radically increasing over a period, despite past experiences of the toll of deaths caused by many infectious diseases. The fluctuations in the life expectancy over a period were due to a number of infectious diseases such as HIV and influenza in India. In the USA and Liberia, previous epidemics such as the 1918 influenza pandemic and the 2014 Ebola virus outbreak resulted in the decline in life expectancy by 11.8 years and 1.6–5.6 years, respectively (27). The epidemiological transition can be controlled by reducing the deaths from endemic diseases through vaccination and immunization programs, better sanitation, better housing, social welfare programs, and improved health system practices through technological advancements and biomedical research.

## Conclusion

Life expectancy is primarily a public health measure that can be compared across the nations and large subpopulation. It has been used as an indicator for both economic success and the effectiveness of medical care. The COVID−19 pandemic is the recent experience of high morbidity and mortality that will reverse the secular trend of increasing life expectancy, resulting in a drop in period life expectancy. This study found that there is a decrease in life expectancy by 43 days (0.12 years) due to COVID−19 during 2020. Even though mortality due to COVID−19 was high, factors such as lockdown, vaccination, and accidents also had an influence on mortality. Thus, there is a need to assess the impact of COVID−19 on life expectancy in future. Further, a comprehensive pandemic preparedness aimed at more resilient health systems, interventions on the reduction of premature mortality, and timely monitoring of excess mortality will help future policy interventions.

## Data availability statement

The datasets presented in this study can be found in online repositories. The names of the repository/repositories and accession number(s) can be found in the article/Supplementary material.

## Author contributions

MM, PS, and NK were responsible for conceptualization of the study, data collection, supervision, project administration, and formal analysis. MM, PS, YA, and NK were responsible for the methodology. MM, PS, and YA were responsible for data collection. MM, NK, and CP were responsible for the original draft preparation. MM, PS, YA, NK, and CP were responsible for review and editing of the manuscript. All authors have read and agreed to publish the manuscript.

## Conflict of interest

The authors declare that the research was conducted in the absence of any commercial or financial relationships that could be construed as a potential conflict of interest.

## Publisher's note

All claims expressed in this article are solely those of the authors and do not necessarily represent those of their affiliated organizations, or those of the publisher, the editors and the reviewers. Any product that may be evaluated in this article, or claim that may be made by its manufacturer, is not guaranteed or endorsed by the publisher.
