# Supplementary material for: A national-level analysis of life expectancy associated with the COVID-19 pandemic in India
Source: Front Public Health. 2022 Oct 18;10:1000933. doi: 10.3389/fpubh.2022.1000933 (PMC9623254; doi:10.3389/fpubh.2022.1000933)
Supplement: Supplementary file 1 [file Data_Sheet_1.docx]

**Supplementary**

**A national-level analysis of life expectancy associated with COVID-19 pandemic in India**

M Muniyandi,^1^ Pravin Kumar Singh,^1^ Yamini Aanandh,^1^ N Karikalan^2^ Padmapriyadarsini C^3^

^1^ Department of Health Economics, ICMR-National Institute for Research in Tuberculosis Chennai, India

^2^ Department of Socio-Behavioural Research, ICMR-National Institute for Research in Tuberculosis Chennai, India

^3^ Director & Department of Clinical Research, ICMR-National Institute for Research in Tuberculosis Chennai, India

**S-Table 1. General Abridged annual life table for the Indian population, 2020-2021**

| **Age** | **m(x,n)** | **q(x,n)** | **l(x)** | **d(x,n)** | **L(x,n)** | **S(x,n)** | **T(x)** | **e(x)** | **a(x,n)** |
| --- | --- | --- | --- | --- | --- | --- | --- | --- | --- |
| 0 - 1 | 0.032912 | 0.032 | 100000 | 3200 | 97230.4 | 0.965242 | 6927591 | 69.28 | 0.1345 |
| 1 - 5 | 0.001921 | 0.00765 | 96800 | 740.52 | 385390.6 | 0.993404 | 6830361 | 70.56 | 1.556584 |
| 5 - 10 | 0.000717 | 0.00358 | 96059.48 | 343.8929 | 479437.7 | 0.996704 | 6444970 | 67.09 | 2.500000 |
| 10 - 15 | 0.000603 | 0.00301 | 95715.59 | 288.1039 | 477857.7 | 0.996292 | 5965533 | 62.33 | 2.500000 |
| 15 - 20 | 0.000948 | 0.00473 | 95427.48 | 451.372 | 476085.9 | 0.99418 | 5487675 | 57.51 | 2.670321 |
| 20 - 25 | 0.001379 | 0.00687 | 94976.11 | 652.4859 | 473315 | 0.992662 | 5011589 | 52.77 | 2.600591 |
| 25 - 30 | 0.001558 | 0.00776 | 94323.63 | 731.9513 | 469842 | 0.991283 | 4538274 | 48.11 | 2.573383 |
| 30 - 35 | 0.001991 | 0.00991 | 93591.67 | 927.4935 | 465746.4 | 0.988308 | 4068432 | 43.47 | 2.615151 |
| 35 - 40 | 0.002762 | 0.01372 | 92664.18 | 1271.353 | 460300.9 | 0.984071 | 3602686 | 38.88 | 2.624572 |
| 40 - 45 | 0.003723 | 0.01845 | 91392.83 | 1686.198 | 452968.8 | 0.977955 | 3142385 | 34.38 | 2.630541 |
| 45 - 50 | 0.005364 | 0.02649 | 89706.63 | 2376.329 | 442983.1 | 0.966203 | 2689416 | 29.98 | 2.664460 |
| 50 - 55 | 0.008649 | 0.04239 | 87330.3 | 3701.931 | 428011.7 | 0.947917 | 2246433 | 25.72 | 2.666130 |
| 55 - 60 | 0.012984 | 0.06299 | 83628.37 | 5267.751 | 405719.6 | 0.923189 | 1818421 | 21.74 | 2.641828 |
| 60 - 65 | 0.019454 | 0.09299 | 78360.62 | 7286.754 | 374556.1 | 0.885912 | 1412702 | 18.03 | 2.633098 |
| 65 - 70 | 0.029878 | 0.13949 | 71073.87 | 9914.093 | 331823.9 | 0.82608 | 1038146 | 14.61 | 2.625051 |
| 70 - 75 | 0.047803 | 0.21425 | 61159.77 | 13103.48 | 274113.1 | 0.745046 | 706321.7 | 11.55 | 2.581886 |
| 75 - 80 | 0.071393 | 0.3034 | 48056.29 | 14580.28 | 204226.9 | 0.638348 | 432208.6 | 8.99 | 2.527171 |
| 80 - 85 | 0.111212 | 0.4331 | 33476.01 | 14498.46 | 130367.9 | 0.501212 | 227981.7 | 6.81 | 2.447165 |
| 85 - 90 | 0.168461 | 0.58003 | 18977.55 | 11007.55 | 65341.9 | 0.352374 | 97613.77 | 5.14 | 2.315855 |
| 90 - 95 | 0.247683 | 0.71554 | 7970.002 | 5702.855 | 23024.8 | 0.286537 | 32271.87 | 4.05 | 2.049686 |
| 95 -100 | 0.245174 | ... | 2267.147 | 2267.147 | 9247.076 | ... | 9247.076 | 4.08 | 4.078728 |

**Age=**the initial age of the age interval; **m(x,n)=**death rate for the age interval; **q(x,n)=**probability of an individual age x dying before the end of the age interval (x, x+n). **l(x)=**number of survivors at age x; **d(x,n)=**number of deaths in age interval (x, x+n); **L(x,n)=**number of person-years lived in age interval (x, x+n); **S(x,n)=**the proportion of the life table population in age group (x, x+n) who are alive n year later; **T(x)=**number of person-years lived at ages x and older; **e(x)=**expectation of life at age x; **a(x,n)=**average number of years lived in the age interval (x, x+n)

**S-Table 2. Abridged annual life table with the impact of COVID-19 for the Indian population, up to June 2021**

| **Age** | **m(x,n)** | **q(x,n)** | **l(x)** | **d(x,n)** | **L(x,n)** | **S(x,n)** | **T(x)** | **e(x)** | **a(x,n)** |
| --- | --- | --- | --- | --- | --- | --- | --- | --- | --- |
| 1 - 5 | 0.001924 | 0.007659 | 96799.84 | 741.3458 | 385387.9 | 0.993397 | 6818454 | 70.44 | 1.556579 |
| 5 - 10 | 0.000718 | 0.003585 | 96058.49 | 344.3851 | 479431.5 | 0.996699 | 6433066 | 66.97 | 2.500000 |
| 10 - 15 | 0.000604 | 0.003016 | 95714.11 | 288.72 | 477848.7 | 0.996281 | 5953634 | 62.2 | 2.500000 |
| 15 - 20 | 0.000952 | 0.004747 | 95425.39 | 452.9832 | 476071.6 | 0.994166 | 5475786 | 57.38 | 2.670333 |
| 20 - 25 | 0.001382 | 0.006885 | 94972.4 | 653.9243 | 473294.1 | 0.992609 | 4999714 | 52.64 | 2.602317 |
| 25 - 30 | 0.001577 | 0.007853 | 94318.48 | 740.6471 | 469796.1 | 0.991189 | 4526420 | 47.99 | 2.574746 |
| 30 - 35 | 0.002009 | 0.009999 | 93577.83 | 935.6768 | 465656.7 | 0.988219 | 4056624 | 43.35 | 2.61404 |
| 35 - 40 | 0.002781 | 0.013812 | 92642.16 | 1279.609 | 460170.9 | 0.983959 | 3590967 | 38.76 | 2.624332 |
| 40 - 45 | 0.003752 | 0.018597 | 91362.55 | 1699.09 | 452789.4 | 0.977698 | 3130796 | 34.27 | 2.632062 |
| 45 - 50 | 0.005442 | 0.026868 | 89663.46 | 2409.102 | 442691.3 | 0.965796 | 2678007 | 29.87 | 2.664695 |
| 50 - 55 | 0.008735 | 0.042804 | 87254.36 | 3734.792 | 427549.4 | 0.947475 | 2235315 | 25.62 | 2.664554 |
| 55 - 60 | 0.013083 | 0.063458 | 83519.56 | 5300.022 | 405092.4 | 0.922755 | 1807766 | 21.64 | 2.640496 |
| 60 - 65 | 0.019543 | 0.093391 | 78219.54 | 7305.037 | 373801.1 | 0.885448 | 1402674 | 17.93 | 2.632235 |
| 65 - 70 | 0.030009 | 0.140063 | 70914.5 | 9932.497 | 330981.3 | 0.825359 | 1028872 | 14.51 | 2.624841 |
| 70 - 75 | 0.048035 | 0.215179 | 60982.01 | 13122.04 | 273178.4 | 0.743839 | 697891.2 | 11.44 | 2.581804 |
| 75 - 80 | 0.071845 | 0.305036 | 47859.97 | 14598.99 | 203200.6 | 0.636086 | 424712.8 | 8.87 | 2.527279 |
| 80 - 85 | 0.112316 | 0.436463 | 33260.98 | 14517.2 | 129253.1 | 0.496316 | 221512.2 | 6.66 | 2.447735 |
| 85 - 90 | 0.171881 | 0.58826 | 18743.78 | 11026.21 | 64150.37 | 0.340468 | 92259.11 | 4.92 | 2.318343 |
| 90 - 95 | 0.261954 | 0.741343 | 7717.569 | 5721.364 | 21841.14 | 0.222977 | 28108.74 | 3.64 | 2.072952 |
| 95 -100 | 0.318496 | ... | 1996.205 | 1996.205 | 6267.6 | ... | 6267.6 | 3.14 | 3.139758 |

**Age=**the initial age of the age interval; **m(x,n)=**death rate for the age interval; **q(x,n)=**probability of an individual age x dying before the end of the age interval (x, x+n). **l(x)=**number of survivors at age x; **d(x,n)=**number of deaths in age interval (x, x+n); **L(x,n)=**number of person-years lived in age interval (x, x+n); **S(x,n)=**the proportion of the life table population in age group (x, x+n) who are alive n year later; **T(x)=**number of person-years lived at ages x and older; **e(x)=**expectation of life at age x; **a(x,n)=**average number of years lived in the age interval (x, x+n)

**S-Table 3. Comparison of life expectancy during pre and post COVID-19 in India**

**(In Years)**

| Age group | Without COVID-19 | With COVID-19 | Difference |
| --- | --- | --- | --- |
| 0-1 | 69.28 | 69.16 | 0.12 |
| 01-05 | 70.56 | 70.44 | 0.12 |
| 05-10 | 67.09 | 66.97 | 0.12 |
| 10-15 | 62.33 | 62.2 | 0.12 |
| 15-20 | 57.51 | 57.38 | 0.12 |
| 20-25 | 52.77 | 52.64 | 0.12 |
| 25-30 | 48.11 | 47.99 | 0.12 |
| 30-35 | 43.47 | 43.35 | 0.12 |
| 35-40 | 38.88 | 38.76 | 0.12 |
| 40-45 | 34.38 | 34.27 | 0.12 |
| 45-50 | 29.98 | 29.87 | 0.11 |
| 50-55 | 25.72 | 25.62 | 0.11 |
| 55-60 | 21.74 | 21.64 | 0.1 |
| 60-65 | 18.03 | 17.93 | 0.1 |
| 65-70 | 14.61 | 14.51 | 0.1 |
| 70-75 | 11.55 | 11.44 | 0.1 |
| 75-80 | 8.99 | 8.87 | 0.12 |
| 80-85 | 6.81 | 6.66 | 0.15 |
| 85-90 | 5.14 | 4.92 | 0.22 |
| 90-95 | 4.05 | 3.64 | 0.41 |
| 95-100 | 4.08 | 3.14 | 0.94 |

**S-Table 4. Comparison of life expectancy during pre and post COVID-19 in India**

**(In Days)**

| Age | Without COVID-19 | With COVID-19 | Difference |
| --- | --- | --- | --- |
| 0-1 | 25286 | 25242 | 43 |
| 01-05 | 25755 | 25710 | 45 |
| 05-10 | 24489 | 24444 | 45 |
| 10t-15 | 22749 | 22704 | 45 |
| 15-20 | 20990 | 20945 | 45 |
| 20-25 | 19260 | 19215 | 45 |
| 25-30 | 17562 | 17517 | 45 |
| 30-35 | 15867 | 15823 | 44 |
| 35-40 | 14191 | 14148 | 43 |
| 40-45 | 12550 | 12508 | 42 |
| 45-50 | 10943 | 10902 | 41 |
| 50-55 | 9389 | 9351 | 38 |
| 55-60 | 7937 | 7900 | 36 |
| 60-65 | 6580 | 6545 | 35 |
| 65-70 | 5331 | 5296 | 36 |
| 70-75 | 4215 | 4177 | 38 |
| 75-80 | 3283 | 3239 | 44 |
| 80-85 | 2486 | 2431 | 55 |
| 85-90 | 1877 | 1797 | 81 |
| 90-95 | 1478 | 1329 | 149 |
| 95-100 | 1489 | 1146 | 343 |

**S-Table 5. Abridged annual life table with the impact of COVID-19 for the Indian population, before and during COVID-19**

| Age Group | Before COVID-19 | 2020 | | 2021 | | 2022 |
| --- | --- | --- | --- | --- | --- | --- |
|  |  | Jan-June | July-Dec | Jan-June | July-Dec | Jan-June |
| 0 - 1 | 69.28 | 69.27 | 69.20 | 69.12 | 69.23 | 69.25 |
| 1 - 5 | 70.56 | 70.55 | 70.48 | 70.40 | 70.51 | 70.54 |
| 5 - 10 | 67.09 | 67.08 | 67.01 | 66.93 | 67.04 | 67.07 |
| 10 - 15 | 62.33 | 62.32 | 62.24 | 62.16 | 62.28 | 62.30 |
| 15 - 20 | 57.51 | 57.50 | 57.43 | 57.34 | 57.46 | 57.48 |
| 20 - 25 | 52.77 | 52.76 | 52.69 | 52.60 | 52.72 | 52.74 |
| 25 - 30 | 48.11 | 48.10 | 48.03 | 47.95 | 48.06 | 48.09 |
| 30 - 35 | 43.47 | 43.46 | 43.39 | 43.31 | 43.42 | 43.44 |
| 35 - 40 | 38.88 | 38.87 | 38.80 | 38.72 | 38.83 | 38.85 |
| 40 - 45 | 34.38 | 34.37 | 34.31 | 34.23 | 34.34 | 34.36 |
| 45 - 50 | 29.98 | 29.97 | 29.91 | 29.83 | 29.93 | 29.96 |
| 50 - 55 | 25.72 | 25.71 | 25.65 | 25.58 | 25.68 | 25.70 |
| 55 - 60 | 21.74 | 21.74 | 21.68 | 21.61 | 21.70 | 21.72 |
| 60 - 65 | 18.03 | 18.02 | 17.97 | 17.90 | 17.99 | 18.01 |
| 65 - 70 | 14.61 | 14.60 | 14.54 | 14.48 | 14.57 | 14.58 |
| 70 - 75 | 11.55 | 11.54 | 11.48 | 11.41 | 11.51 | 11.53 |
| 75 - 80 | 8.99 | 8.98 | 8.91 | 8.84 | 8.94 | 8.97 |
| 80 - 85 | 6.81 | 6.80 | 6.71 | 6.61 | 6.75 | 6.78 |
| 85 - 90 | 5.14 | 5.12 | 5.00 | 4.85 | 5.05 | 5.09 |
| 90 - 95 | 4.05 | 4.01 | 3.78 | 3.52 | 3.88 | 3.96 |
| 95 -100 | 4.08 | 4.00 | 3.48 | 2.81 | 3.71 | 3.88 |

**S-Figure-1. Mortality due to COVID-19 for different age groups in India**

**S-Figure-2. Estimated life expectancy from July 2019 to June 2022 in India**

**S-Figure-3. The life expectancy at birth in India from 1950 to 2021**
